# *In Situ* Actuators with Gallium Liquid Metal Alloys and Polypyrrole-Coated Electrodes

**DOI:** 10.1021/acsami.2c17906

**Published:** 2023-02-08

**Authors:** Sagar Bhagwat, Andreas Goralczyk, Manuel Luitz, Lathif Sharieff, Sebastian Kluck, Ahmed Hamza, Niloofar Nekoonam, Frederik Kotz-Helmer, Pegah Pezeshkpour, Bastian E. Rapp

**Affiliations:** † Laboratory of Process Technology, NeptunLab, Department of Microsystems Engineering (IMTEK), University of Freiburg, Georges-Köhler-Allee 103, 79110 Freiburg, Germany; ‡ Freiburg Materials Research Center (FMF), University of Freiburg, Stefan-Meier-Straße 21, 79104 Freiburg, Germany; § FIT Freiburg Center of Interactive Materials and Bioinspired Technologies, University of Freiburg, Georges-Köhler-Allee 105, 79110 Freiburg, Germany

**Keywords:** Galinstan, alloying, actuation, polypyrrole, 3D printing

## Abstract

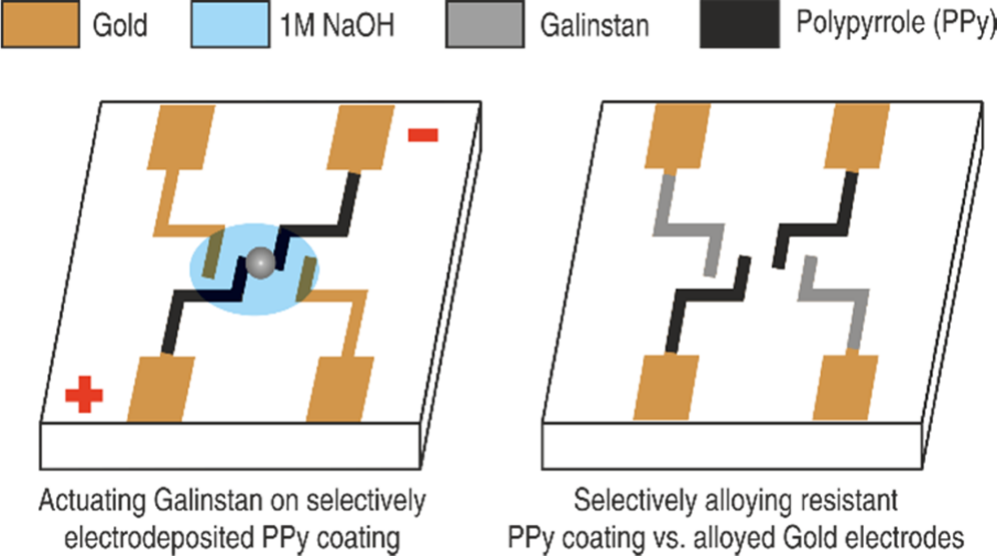

Gallium liquid metal alloys (GLMAs) such as Galinstan and gallium–indium eutectic (EGaIn) are interesting materials due to their high surface tensions, low viscosities, and electrical conductivities comparable to classical solid metals. They have been used for applications in microelectromechanical systems (MEMS) and, more recently, liquid metal microfluidics (LMMF) for setting up devices like actuators. However, their high tendency to alloy with the most common metals used for electrodes such as gold (Au), platinum (Pt), titanium (Ti), nickel (Ni), and tungsten–titanium (WTi) is a major problem limiting the scaleup and applicability, e.g., liquid metal actuators. Stable electrodes are key elements for many applications and thus, the lack of an electrode material compatible with GLMAs is detrimental for many potential application scenarios. In this work, we study the effect of actuating Galinstan on various solid metal electrodes and present an electrode protection methodology that, first, prevents alloying and, second, prevents electrode corrosion. We demonstrate reproducible actuation of GLMA segments in LMMF, showcasing the stability of the proposed protective coating. We investigated a range of electrode materials including Au, Pt, Ti, Ni, and WTi, all in aqueous environments, and present the resulting corrosion/alloying effects by studying the interface morphology. Our proposed protective coating is based on a simple method to electrodeposit electrically conductive polypyrrole (PPy) on the electrodes to provide a conductive alloying-barrier layer for applications involving direct contact between GLMAs and electrodes. We demonstrate the versatility of this approach by direct three-dimensional (3D) printing of a 500 μm microfluidic chip on a set of electrodes onto which PPy is electrodeposited *in situ* for actuation of Galinstan plugs. The developed protection protocol will provide a generic, widely applicable strategy to protect a wide range of electrodes from alloying and corrosion and thus form a key element in future applications of GLMAs.

## Introduction

Gallium liquid metal alloys (GLMAs) such as gallium–indium eutectic (EGaIn; 75 wt % Ga, 25 wt % In; melting point: 14.2 °C) and Galinstan (68.5 wt % Ga, 21 wt % In, 10 wt % Sn; melting point: 13.2 °C) have recently seen increasing interest for a wide range of applications due to their interesting combination of properties stemming from the fact that they are both metallic in nature (and thus electrical conductivities similar to bulk metals) and are liquids with very low viscosity (about twice the viscosity of water), combined with their very high surface tension, again, similar to bulk metals (∼600 to 700 mNm^–1^). In contrast to other liquid metals, e.g., mercury (Hg), they have negligible toxicity and feature extremely low vapor pressures (<10^–6^ Pa).^1,2^ This offers many advantages and GLMAs have been used in many applications including self-healing and flexible electronics,^3^ actuators,^4^ microfluidics,^5,6^ microswitches,^7^ droplet generators,^8−10^ reconfigurable devices,^11,12^ shape-memory,^13^ 3D printing,^14−16^ catalysis,^17,18^ and nanotechnology.^19^ GLMAs have the tendency to form a nanometer-thick oxide skin on the surface that not only provides liquid-like properties due to lowering of the surface tension but also hampers the performance of GLMAs, especially when the oxide skin selectively wets to metal electrodes (ME), resulting in embrittlement and alloying of the electrode material owing to intermetallic bonding between GLMAs and ME.^20−22^ The oxide skin can be removed by acids and bases such as hydrochloric acid (HCl)^23^ or sodium hydroxide (NaOH)^24^ and several electrolytes like sodium chloride (NaCl).^25−27^ However, the continuously regenerating oxide skin is a nuisance for applications that involve localized actuation in microstructures.

Functionalizing the GLMA oxide skin or the surface of conductive electrodes with additional alloying-barrier layers is necessary to achieve proper liquid metal (LM) motion upon actuation on MEs. Various techniques including using phosphonic acids,^11^ colloidal nanoparticle suspensions of metal oxides such as WO_3_, TiO_2_, MoO_3_, and In_2_O_3_,^28^ functional diblock polymer surfactants,^29^ single-walled carbon nanotubes (SWCNTs),^30^ graphene,^31^ diamond coatings,^32^ carbon ink,^33^ poly(vinylpyrrolidone),^34^ and conductive polymers like poly(3,4-ethylenedioxythiophene) polystyrene sulfonate (PEDOT:PSS)^35^ and polypyrrole (PPy)^36^ have been reported to address these problems. Almost all of these techniques involve multiple processing steps that alter the properties of GLMAs and limit their applicability and scalability. An elegant solution to this problem is the introduction of an alloying-barrier layer on the conductive electrodes. Oh et al. presented an alloy diffusion barrier layer based on graphene patterned on SWCNTs to combine the electrical conductivity with the flexibility of the nanotubes.^30^ Although well adopted for stretchable electronics, the process involves multiple steps. Ahlberg et al. have also shown excellent diffusion barrier properties of graphene on aluminum films against Galinstan; however, the stability of graphene coating under actuation conditions was not studied.^37^ Handschuh-Wang et al. have shown boron-doped conductive and nonconductive diamond coatings on titanium and silicon substrates with excellent resistance to GLMAs. However, the intricate synthesis procedure involves a diamond nanoparticle seeding step followed by hot-filament chemical vapor deposition (HFCVD).^32^ Shin et al. successfully tested the effect of aluminum electrodes coated with solution-processed PEDOT:PSS/graphene oxide composites for fluidic interconnections in flexible electronics.^35^ However, the coating synthesis is process-intensive and difficult to scale up due to the challenges involved in microstructuring aluminum. Joshipura et al. presented a spray-coating technique using a commercially available superomniphobic coating (NeverWet) on various substrates to enhance surface roughness and prevent adhesion of GLMAs.^38^ However, as masking is difficult in applying these coatings, the layer usually covers the entire substrate surface and limits LM actuation under applied electrical potentials. In another study, Geddis et al. investigated the corrosive behavior of Galinstan with solid metals including aluminum, copper, nickel–chromium, and brass, where nickel–chromium alloys exhibited alloying-barrier properties.^39^ However, no study was conducted to analyze the behavior of actuating Galinstan in conductive solutions on these solid metals. Most of these studies indicate the lack of a simple method to fabricate electrodes with an alloying-barrier layer that resists embrittlement, crucially for actuation of GLMAs in the presence of an electrically conductive medium. In addition to the choice of the nonalloying electrode material in GLMA actuation, choosing the right fabrication technique also plays a significant role. For fabricating electrodes in microchannels, various techniques exist including sputtering via physical vapor deposition (PVD) and photolithography,^40−42^ xurography (razor blade writing) and cold lamination,^43^ ink-jet printing,^44^ conductive inks,^14,33^ and organic–inorganic photoresins.^45,46^ Among all of these techniques, integrating PVD-based sputtering and photolithography is the preferred pathway as it combines the wide range of metals that can be sputtered, with the unmatched structuring capabilities from micrometer to millimeter scales via photolithography. However, almost all of the sputtered metal candidates are susceptible to alloying and embrittlement by GLMAs and the selective few that are resistant to alloying have not been studied for LM actuation. An alloying-barrier coating that is conductive and easy to implement on the sputtered and microstructured electrodes solves many challenges in GLMA actuation. One relatively simple way is to functionalize the electrodes by electrodepositing an alloying-barrier layer and a conductive polymer such as PPy. PPy resists alloying and embrittlement, while offering a stable response to electrochemical actuation in acidic or basic mediums. Electrodepositing PPy on electroplated Au electrodes results in a roughened and porous surface, which has been shown to enhance the adhesion of PPy, thus overcoming its tendency to delaminate and allowing long-term stability.^47,48^ Combining sputtering, photolithography, electroplating, and electrodeposition of PPy as the alloying-barrier layer would provide Au electrodes suitable for direct-contact applications in microfluidics, in particular, in liquid metal microfluidics (LMMF).

**Figure 1:**
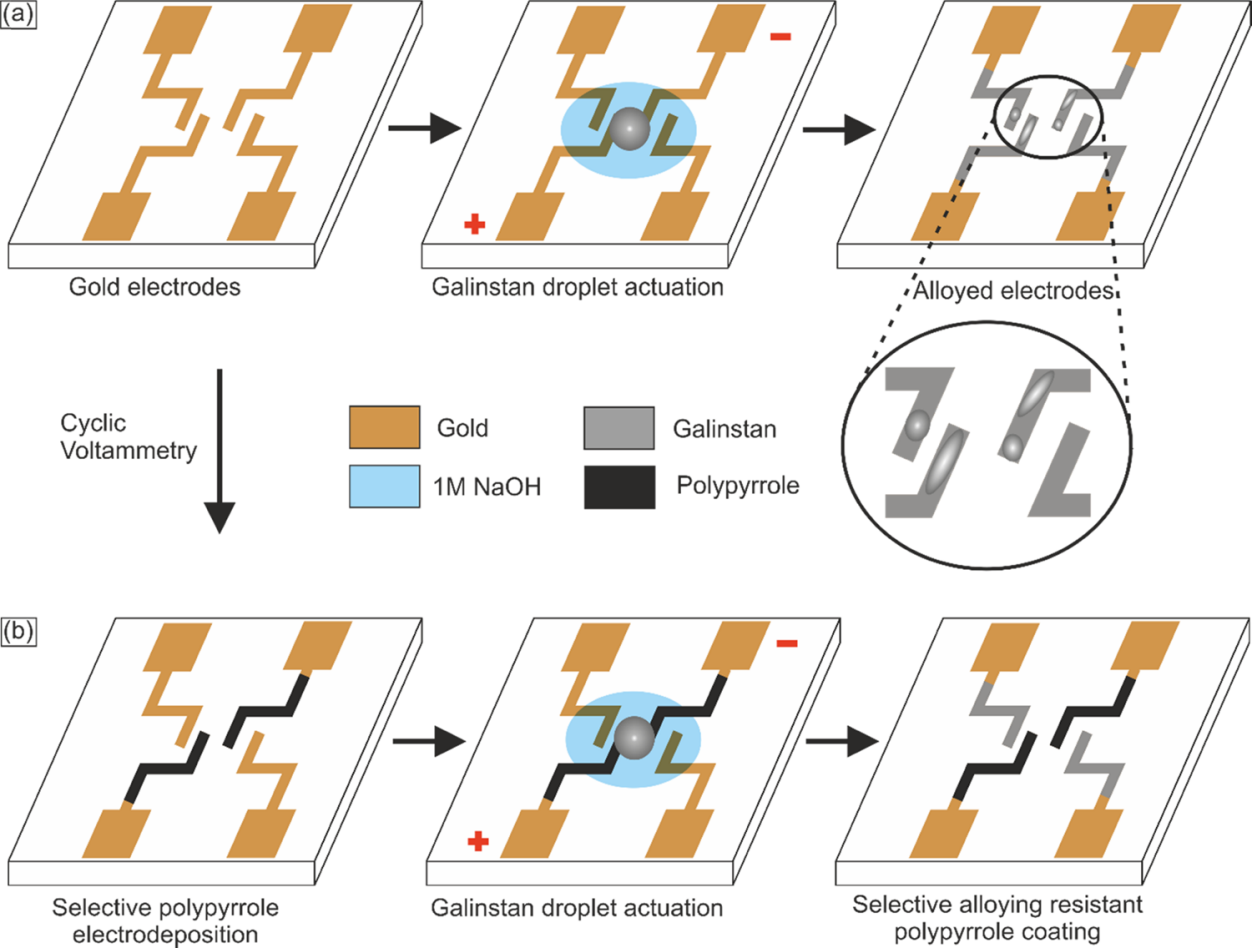
Schematic overview of the concept proposed in this work. (a) Alloying of Au electrodes by actuating a drop of Galinstan at −1 V in 1 M NaOH (inset represents alloying of Galinstan on Au electrodes post actuation). (b) Selective electrodeposition of PPy resulting in alloying-resistant electrodes on actuating a drop of Galinstan at −3 V in 1 M NaOH.

In this work, we studied the effect of actuating Galinstan directly over metal electrodes of Au, Pt, Ti, Ni, and WTi fabricated via sputtering and photolithography and discuss the resulting embrittlement and alloying in 1 M HCl and 1 M NaOH solutions under applied voltages (Figure 1a). We then present a simple way to selectively electrodeposit the alloying-barrier PPy coating on etched Au electrodes for the actuation of Galinstan (Figure 1b). We chose Au as the electrode material because of its excellent stability in acidic and basic media along with its high conductivity that would complement the electrodeposited PPy alloying-barrier layer. We further show the stability of PPy on etched Au electrodes via continuous electrowetting (CEW) experiments in 1 M NaOH in 3D printed microchannels. This work presents a significant step toward a streamlined, fast, and scalable methodology for the generation of high-resolution metal electrodes effectively preventing the alloying of LMs, thus facilitating a wide range of applications in LMMF and microactuator applications.

## Experimental Section

### Materials

Galinstan was purchased and used as received from Strategic Elements (Germany). Sodium hydroxide (NaOH, ≥98%), potassium chloride (KCl, ≥99.5%), hydrochloric acid (HCl, 37%), dimethylsulfoxide (DMSO, 99.5%), and 2-propanol were purchased from Carl Roth (Germany) and used as received. Pyrrole (reagent grade, 98%) was purchased from Sigma-Aldrich (Germany). 3-Methacryloxypropyl-dimethylchlorosilane (MACS) was purchased from abcr (Germany). Nexterion cleanroom glass slides (76 × 52 × 1 mm^3^) were used as substrates and were provided by Schott (Germany). Injekt-F single use syringes of 1 mL capacity were purchased from B. Braun (Germany) and were used as received. The 0.5-in dispensing tips with 0.25 mm interior diameter (Product ID F560015) were purchased from Vieweg (Germany) and used as received. AZ 1518 positive photoresist and AZ 726 MIF (metal ion free) developer were purchased from MicroChemicals GmbH (Germany). LOR 5A (lift-off resist) was purchased from microresist technology GmbH (Germany). The Auroblex 3202 gold electrolyte was purchased from Blendl GmbH, Germany. Potassium iodide (KI, reagent grade) and iodine solution (0.1N) were purchased from Merck Chemicals GmbH (Germany).

### Substrate Preparation via Photolithography

The glass slides were first rinsed with 2-propanol and then 1 mL of LOR 5A, an image reversal resist was drop-casted, and a 1 μm layer was spin-coated for 30 s at 2000 rpm according to the manufacturer’s instructions on a table-top spin-coater (SPIN200i, APT GmbH, Germany). The slides were then prebaked at 150 °C for 1 min on a HP40A programmable laboratory hot-plate by Torrey Pines (MS Scientific Chromatographie-Handel GmbH, Germany) and were allowed to cool to room temperature (RT). AZ 1518 (1–2 mL) was drop-casted and a 1.8 μm layer was spin-coated for 30 s at 4000 rpm followed by a prebaking step at 100 °C for 50 s according to the manufacturer. The desired photomask designed on AutoCAD and printed by Koenen GmbH (Germany) was placed on the spin-coated substrates. The photoresist-coated slides were then exposed at 415 nm for 45 s using a high-pressure mercury lamp Superlite S 04 (Lumatec, Germany) with an exposure dose of 16 mJ cm^–2^. Exposed slides were then developed in AZ 726 MIF developer for 20 s. The slides were rinsed with distilled water (DW) and dried with compressed nitrogen (N_2_). Finally, a post-bake step was performed at 115 °C for 50 s.

### Sputtering of Metals on the Prepared Substrates

The developed slides were placed in a PVD sputtering machine of type FHR Star 100-PentaCo (FHR Anlagenbau GmbH, Germany) in an ISO 5 class 100 cleanroom (IMTEK, University Freiburg, Germany). For all electrode samples except bare titanium (Ti), 20 nm titanium (Ti, 400 W) was sputtered as an adhesion layer with a direct current (DC) generator setup. The 100 nm thin films of gold (Au, 400 W), platinum (Pt, 400 W), nickel (Ni, 300 W), and titanium (Ti, 400 W) were sputtered using the DC generator. Tungsten–titanium alloy (WTi_10_, 400 W) was sputtered with a radiofrequency (RF) generator setup. All of the targets used (100 mm diameter, 6 mm thickness, FHR Anlagenbau GmbH, Germany) were sputtered under 5 × 10^–3^ mbar pressure. Sputtered samples were etched in DMSO for 30 min at 50 °C in a sonication bath, rinsed with 2-propanol, and dried with compressed N_2_.

### Alloying Tests

A 50 μL drop of Galinstan was deposited on metal electrodes, and a drop of 1 M NaOH or 1 M HCl was deposited on top of it. Potential of −1 or −25 V was applied between adjacent electrodes to actuate the droplet via CEW for 15 s. The electrode polarity was changed and the experiment was repeated. Post-actuation, the aqueous solutions and the Galinstan were rinsed off the substrate with DI water and the substrate was subsequently dried with compressed N_2_. Images of the alloyed area were captured on a VHX-6000 digital microscope (Keyence, Germany). The morphology of the alloyed area was studied by capturing micrographs on a scanning electron microscope (SEM) of type Scios 2 DualBeam (Thermo Fisher Scientific, Germany). Energy dispersive X-ray (EDX) spectra were recorded using an Octane Elite EDS System (EDAX, Germany). X-ray diffraction (XRD) patterns were recorded in Braggs–Brentano geometry using a D8 DISCOVER Diffractometer (Bruker, Germany) equipped with Cu Kα radiation, a variable divergence slit, and a LYNXEYE XE-T detector. All scans were carried out in a 2-θ range of 30–60° with a step size of 0.05°.

### Preparation of Gold Electrodes for PPy Electrodeposition

Slides with sputtered Au electrodes were electroplated with a 200 nm Au layer to impart additional roughness on the smooth sputtered Au layer. Auroblex 3202 was used as an electroplating solution at 60 °C, pH 6.6, and 2 mA cm^–2^ to electroplate the given thickness. To ensure PPy adhesion, the electroplated Au electrodes were further etched for 10 s in Au etching solution of KI:I_2_:DW (10:2.5:100 by volume) at RT. The etched slides were rinsed in DW and dried with compressed N_2_.

### Pyrrole Preparation and PPy Electrodeposition

**Figure 2:**
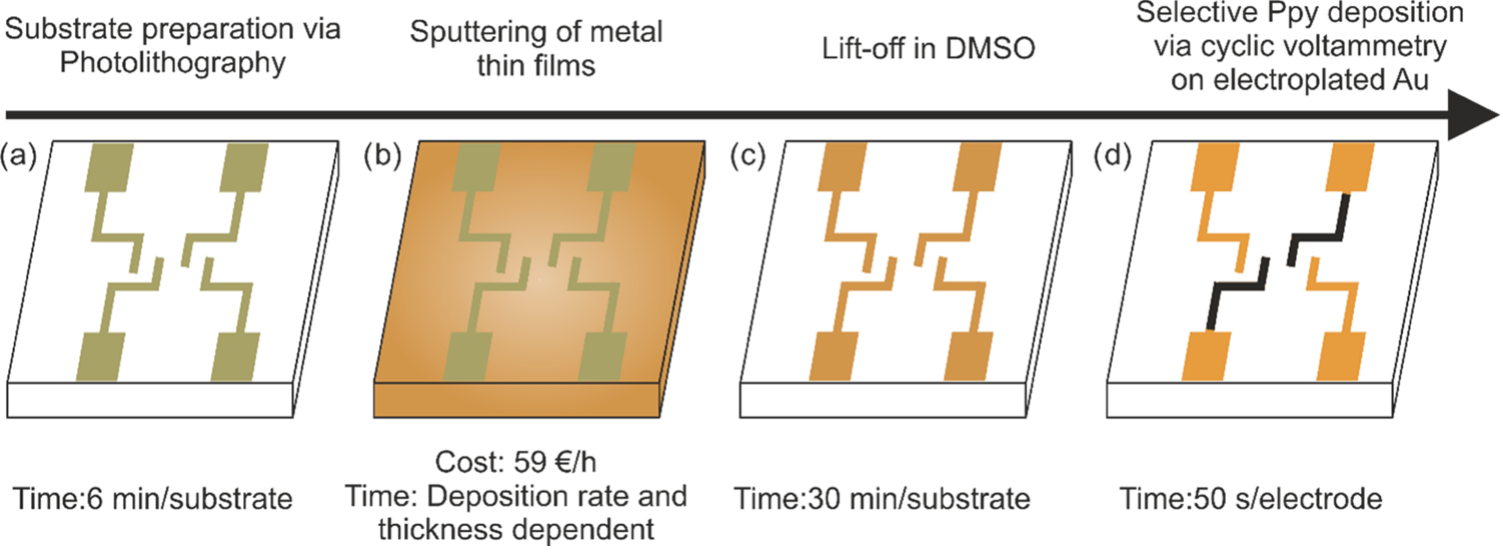
Graphical overview of the major experimental steps (a–d) needed to fabricate PPy-coated electrodes for *in situ* actuator applications with the corresponding time.

Pyrrole was purified by distillation under N_2_ at 70 °C and 70 mbar. Purified pyrrole was stored in a dark bottle purged with N_2_ and sealed with parafilm for further use. PPy was electrochemically deposited on the etched Au electrodes with a solution of 120 mM pyrrole and 100 mM KCl in distilled water via an in-built three-electrode cell consisting of a working electrode (WE), counter electrode (CE), and reference electrode (RE). Cyclic voltammetry (CV) was applied for the three-electrode cell to run voltage scans from 0 to 1000 mV at a rate of 20 mV s^–1^. A graphical overview highlighting the major steps including time and cost for the fabrication of PPy-coated electrodes is included in Figure 2.

### Evaluation of Electrodeposited PPy and Statistical Analysis

The thickness of electrodeposited PPy on etched Au electrodes was analyzed using a White Light Interferometer (WLI) of type NewView 9000 (Zygo). The mean and standard deviation was calculated for the measured thickness profiles recorded via Gwyddion for a sample size of *n* = 11.

### 3D Printing of Microchips on Etched Au Electrodes

A simple closed channel chip was 3D printed on a NanoOne high-resolution printer based on two-photon polymerization (UpNano, Austria) with UpPhoto commercial resin (UpNano, Austria). A 10× air immersion objective (NA 0.4, UPLXAPO10x, Olympus) was used to print the chip on the aforementioned glass slides with electrodes in Vat mode with 80 mW laser power, 5 μm slicing distance, 4.2 μm hatching distance, and 600 mm s^–1^ writing speed. The glass slides with Au electrodes were functionalized with THE MACS protocol as described previously.^49^ Pyrrole (120 mM) and KCl solution (100 mM) were injected into the chip and were subjected to a voltage scan as described above to electrodeposit PPy and conduct CEW experiments with Galinstan in 1 M NaOH.

### CEW Actuation of the Galinstan Plug

To actuate a plug of Galinstan, we used a customized setup that includes a printed circuit board (PCB) consisting of an H-Bridge (L293B) and an Arduino Uno R3 board as elaborated in our earlier work.^10^ We applied a square wave with a delay of 10 ms at 5 V with an applied forward voltage level of 255/255 (5 V) and backward voltage level of 50/255 (1 V). We have included this code in the original Arduino Uno format (.ino) in the Supporting Information.

### Contact Angle Measurements

The wetting behavior of Galinstan on different substrates was characterized using an OCA 15EC CA goniometer (DataPhysics Instruments, Filderstadt, Germany) and evaluated via SCA20 software. The advancing and receding contact angles (ARCA) were measured by first dispensing a 5 μL droplet of Galinstan at 1 μL s^–1^ and contacting the substrate followed by dispensing 10 μL of Galinstan (advancing measurement) and receding till complete removal of Galinstan. The distance between the dispensing needle tip and the substrate was maintained at 0.6 mm. A delay of 30 s was allowed after advancing measurement for Galinstan droplet equilibration. A tangential fitting method was used to evaluate the droplet shape. For each substrate, three ARCA measurements were conducted and after each measurement, the dispensing needle tip was cleaned and about 20 μL of Galinstan was dispensed out to avoid the used oxide from affecting following measurements.

### Electrical Conductivity Measurement

The resistance of all of the substrates used in this work was measured using a digital multimeter (Uni-Trend Multimeter, UT33A) and the corresponding length, width, and thickness were noted down. The conductivity was calculated as the inverse of the measured resistivity.

## Results and Discussion

### Effect of an Actuated Galinstan Droplet on Different Electrodes

**Figure 3:**
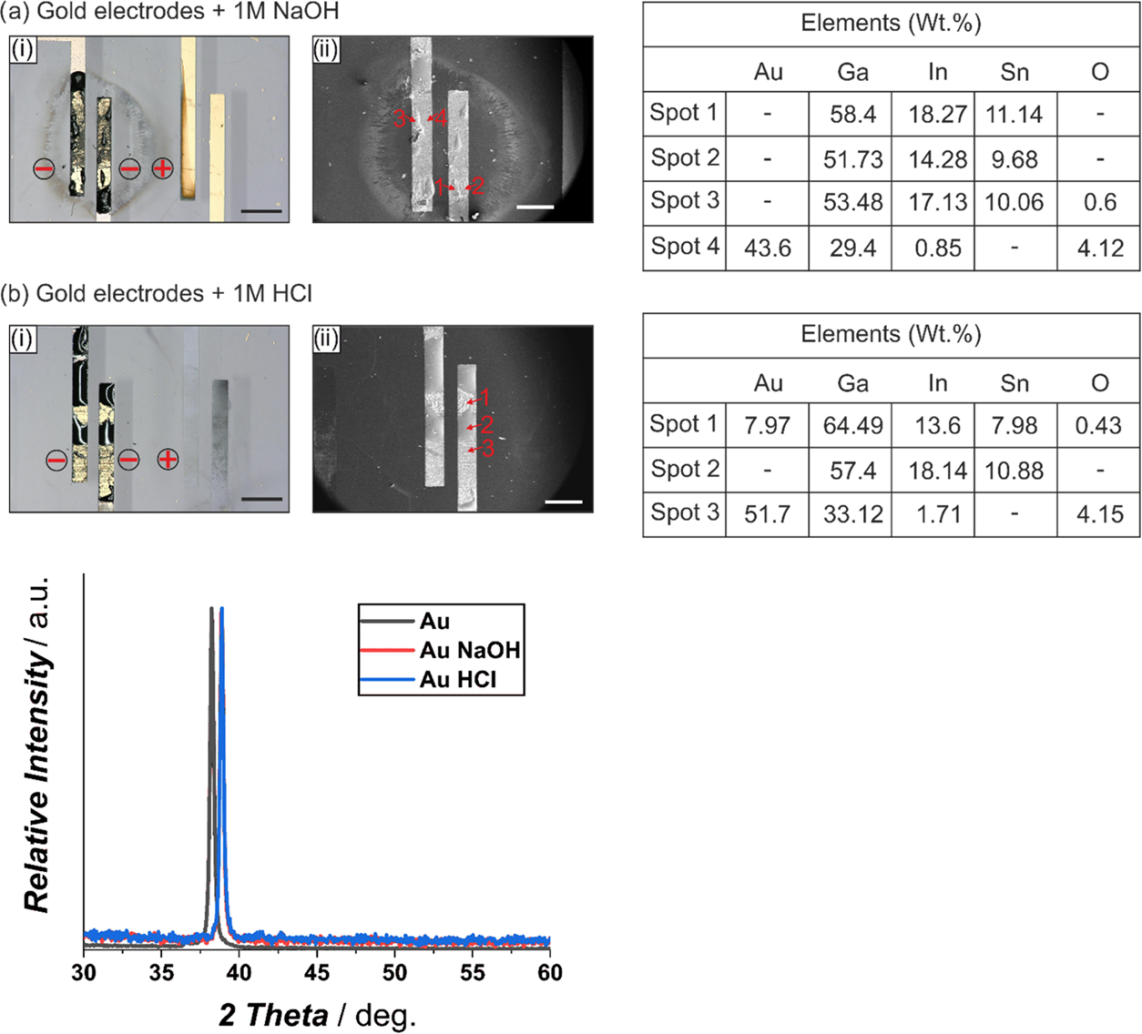
Effect of a Galinstan droplet actuated at −1 V on 100 nm gold electrodes on a 20 nm titanium adhesion layer in the presence of 1 M NaOH (ai, aii) and 1 M HCl (bi, bii), resulting in alloying and electrode surface degradation via optical microscopy images (i, captured at 10° tilt for better visualization; black scale bar: 500 μm), electron microscopy images (ii; white scale bar: 500 μm) with marked spots, the corresponding EDX elemental analysis data, and XRD spectra in comparison with the bare Au electrode. The electrodes used for actuating Galinstan are indicated with – (cathode) and + (anode).

**Figure 4:**
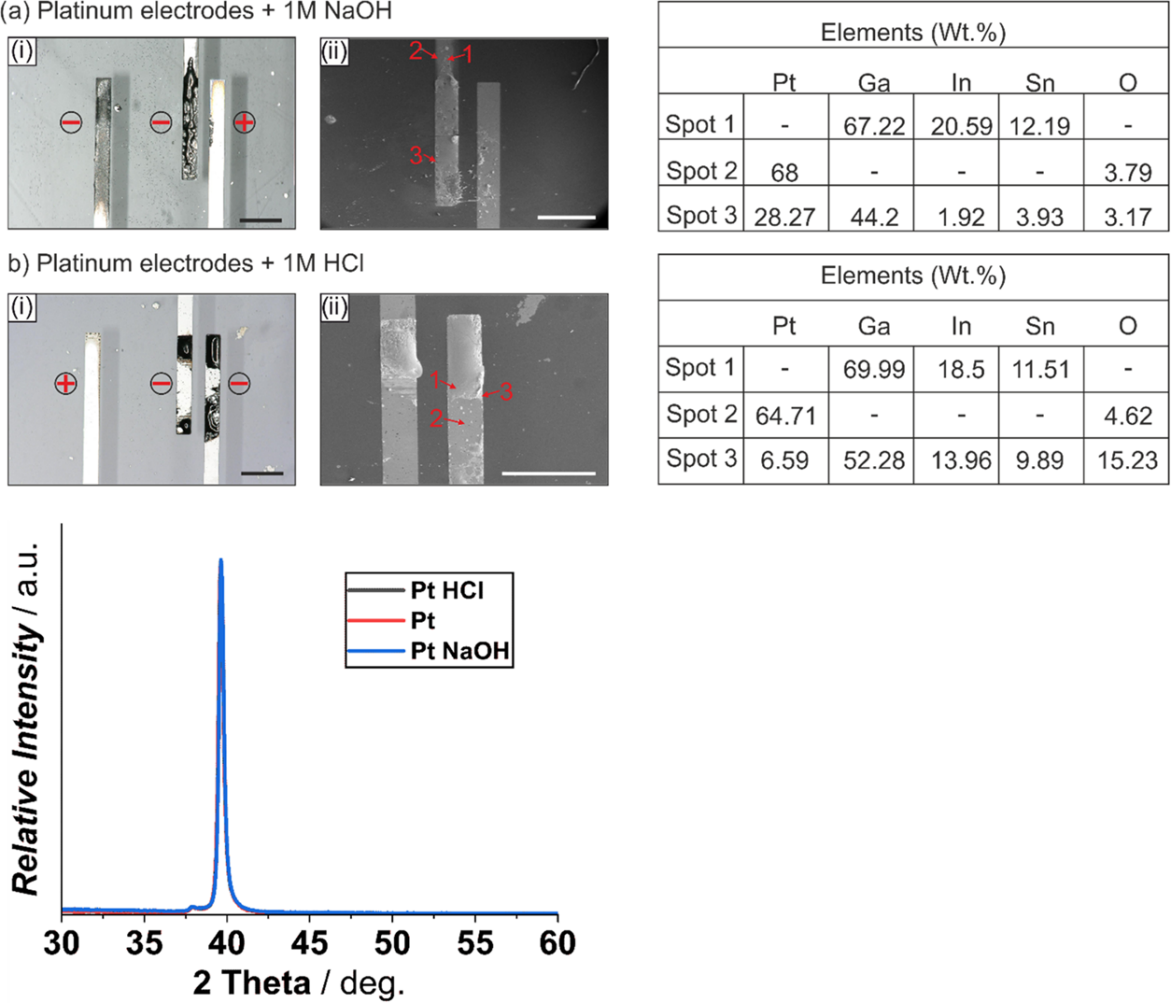
Effect of a Galinstan droplet actuated at −1 V on 100 nm platinum electrodes on a 20 nm titanium adhesion layer in the presence of 1 M NaOH (ai, aii) and 1 M HCl (bi, bii), resulting in alloying and electrode surface degradation via optical microscopy images (i, captured at 10° tilt for better visualization; black scale bar: 500 μm), electron microscopy images (aii—white scale bar: 500 μm, bii—white scale bar: 400 μm) with marked spots, the corresponding EDX elemental analysis data, and XRD spectra in comparison with the bare platinum electrode. The electrodes used for actuating Galinstan are indicated with – (cathode) and + (anode).

**Figure 5:**
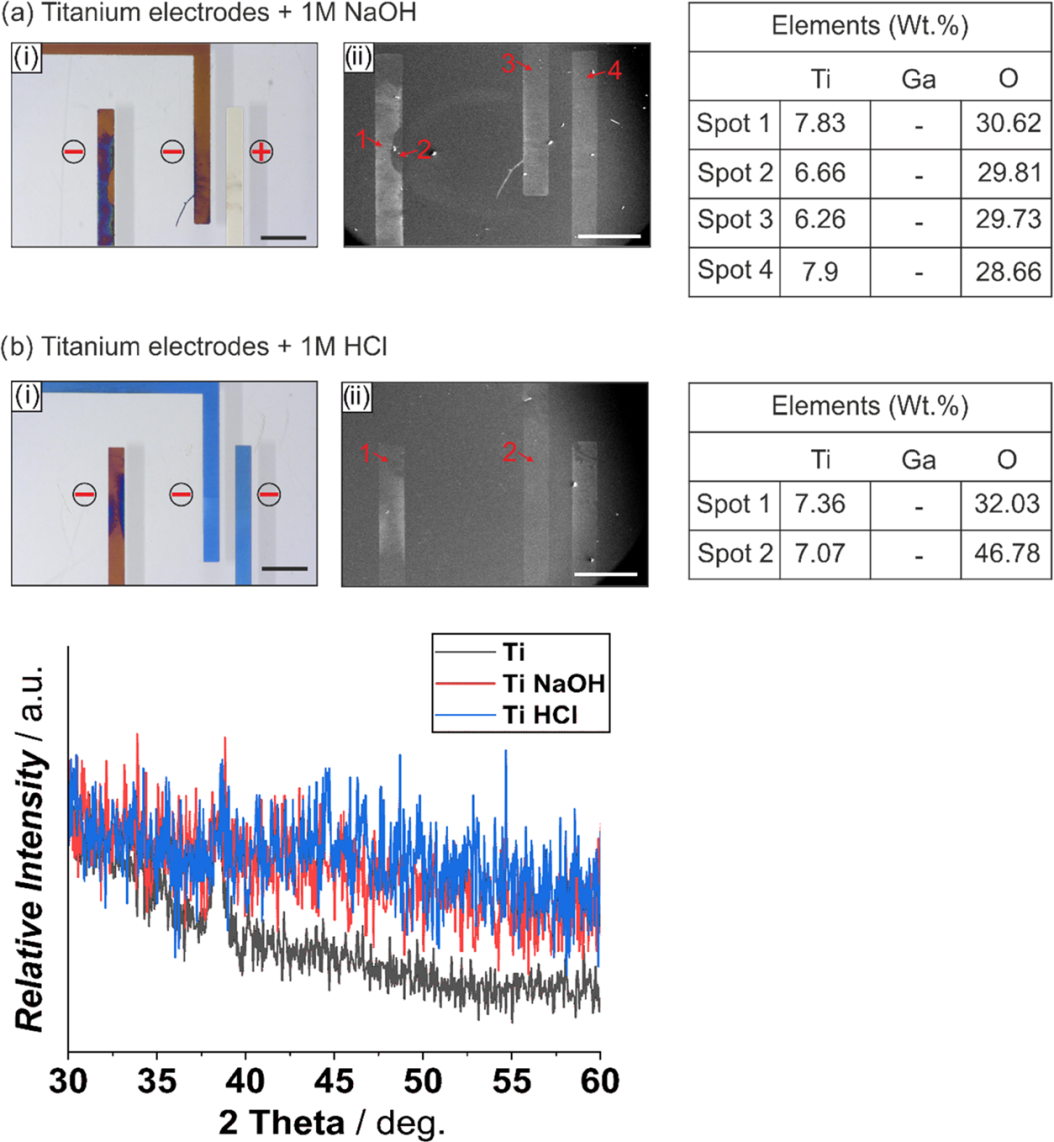
Effect of a Galinstan droplet actuated at −1 V on 100 nm titanium electrodes in the presence of 1 M NaOH (ai, aii) and 1 M HCl (bi, bii), resulting in alloying and electrode surface degradation via optical microscopy images (i, captured at 10° tilt for better visualization; black scale bar: 500 μm), electron microscopy images (ii; white scale bar: 500 μm) with marked spots, the corresponding EDX elemental analysis data, and XRD spectra in comparison with the bare titanium electrode. The electrodes used for actuating Galinstan are indicated with – (cathode) and + (anode).

**Figure 6:**
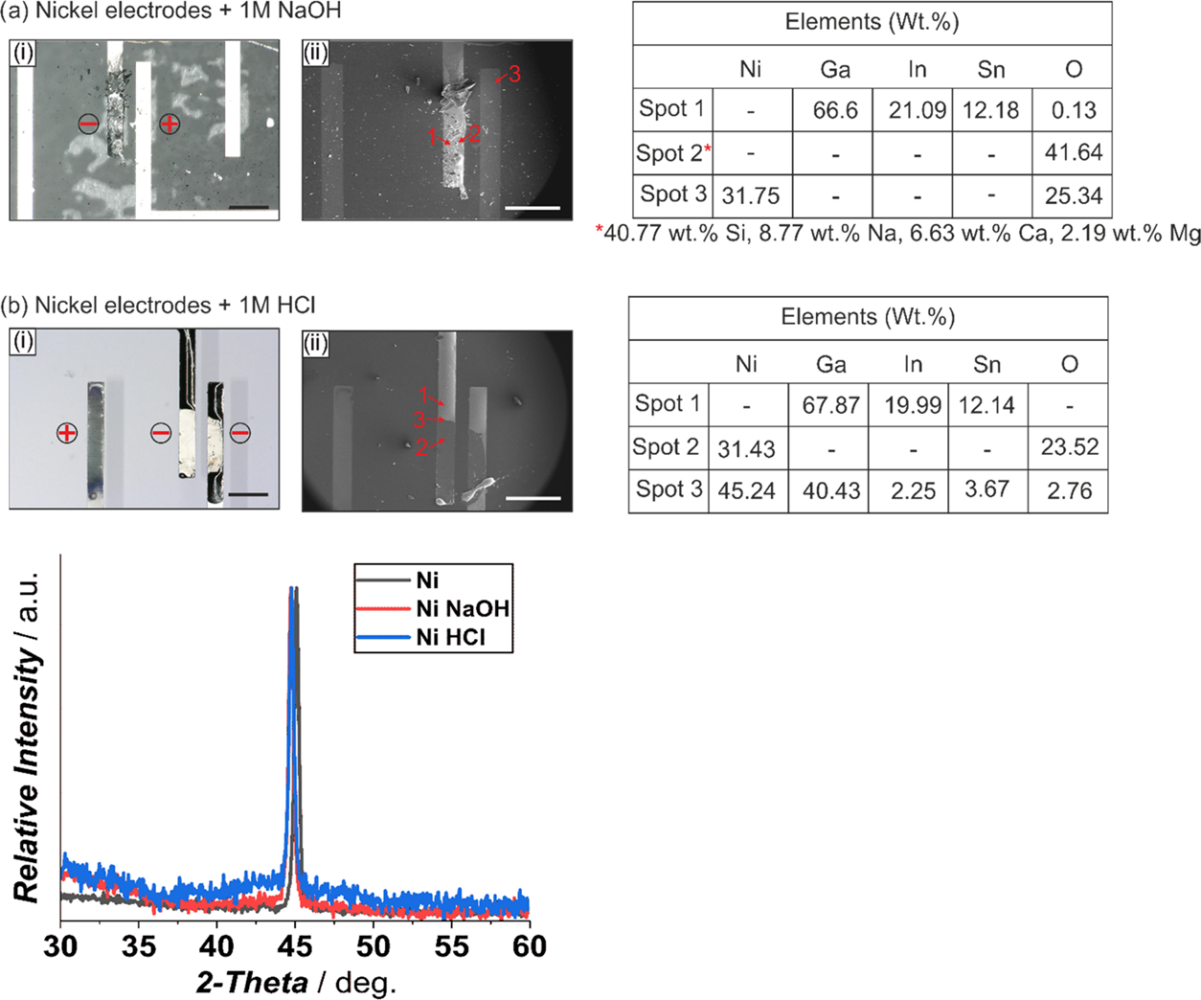
Effect of a Galinstan droplet actuated at −1 V on 100 nm nickel electrodes on a 20 nm Ti adhesion layer in the presence of 1 M NaOH (ai, aii) and 1 M HCl (bi, bii), resulting in alloying and electrode surface degradation via optical microscopy images (i, captured at 10° tilt for better visualization; black scale bar: 500 μm), electron microscopy images (ii; white scale bar: 500 μm) with marked spots, the corresponding EDX elemental analysis data and the XRD spectra in comparison with bare nickel electrode. The electrodes used for actuating Galinstan are indicated with – (cathode) and + (anode).

**Figure 7:**
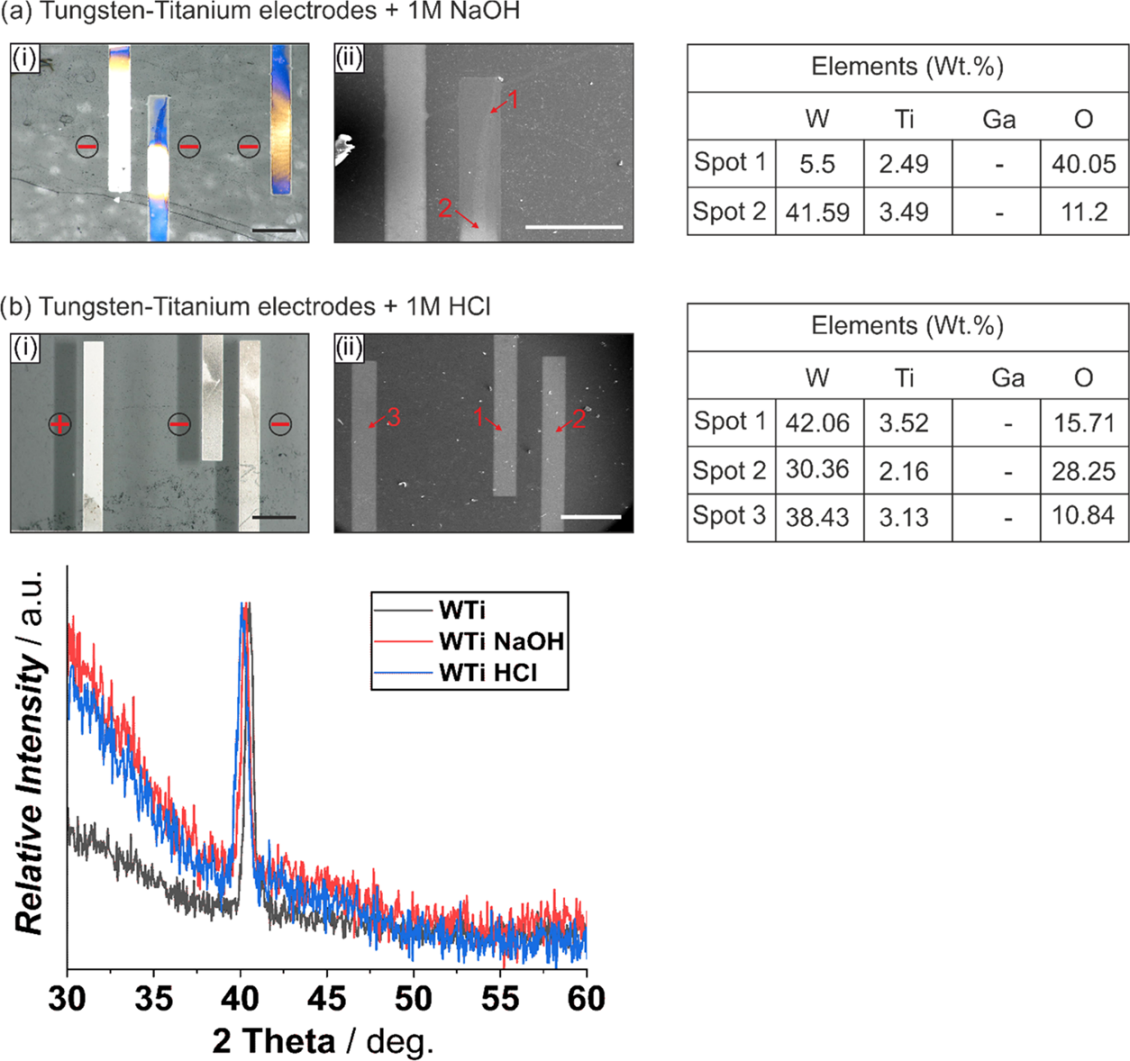
Effect of a Galinstan droplet actuated at −1 V on 100 nm tungsten–titanium electrodes in the presence of 1 M NaOH (ai, aii) and 1 M HCl (bi, bii), resulting in alloying and electrode surface degradation via optical microscopy images (i, captured at 10° tilt for better visualization; black scale bar: 500 μm), electron microscopy images (aii—white scale bar: 400 μm, bii—white scale bar: 500 μm) with marked spots, the corresponding EDX elemental analysis data, and XRD spectra in comparison with the bare WTi electrode. The electrodes used for actuating Galinstan are indicated with – (cathode) and + (anode).

We studied the effect of a Galinstan droplet on various electrode materials by actuating the droplet in the presence of 1 M NaOH and 1 M HCl and analyzing the resulting alloying or electrode degradation. The 100 nm Au electrodes on a 20 nm Ti adhesion layer instantly alloy with Galinstan on actuation in 1 M NaOH at −1 V due to the wetting of Galinstan on Au, which render the electrodes ineffective for further actuation (Figure 3a). The alloyed Galinstan cannot be washed off or removed from the electrodes and results in electrode embrittlement and corrosion. The optical microscopy image (Figure 3ai) shows the strong adhesion of considerable Galinstan residues to the underlying Au electrode. This happened upon the actuation of the Galinstan droplet, where the two electrodes at the right are cathodes and the second electrode from the right is the anode. Elemental spot analysis via EDX (Figure 3aii) with Galinstan post-actuation in 1 M NaOH shows the presence of gallium, indium, and tin, confirming Galinstan for spots 1, 2, and 3, whereas spot 4 indicates an alloy of gold, gallium, and indium in small amounts, with some residual oxide presumably on the surface. Similar alloying and embrittlement of Au electrodes is observed for a Galinstan droplet actuated in 1 M HCl at −1 V as seen in Figure 3bi. In addition to alloying, actuation in HCl results in the release of hydrogen gas at the anode (second electrode from right), which has detrimental effects on the electrode surface. Elemental spot analysis of spot 2 indicates a rich Galinstan phase, whereas spots 1 and 3 are pointing toward a gold gallium alloy, similar to spot 4 in Figure 3bii. The remaining wt % in the respective tables was from carbon, which seems to be an artifact peak. XRD analysis indicates a shift toward larger 2-θ values for the alloyed electrodes in comparison to the bare Au electrode. However, this shift is not significant enough to determine a particular intermetallic alloy for the resulting alloyed samples. Moreover, actuating in 1 M NaOH or 1 M HCl has no effect on the resulting XRD plots as they are identical. This suggests that Au is susceptible to alloying with Galinstan irrespective of the conductive electrolyte. Galinstan actuated in NaOH and HCl on 100 nm Pt electrodes with a 20 nm Ti adhesion layer showed severe alloying and embrittlement, as seen in Figure 4a,b. The alloying effect is instantaneous and can be clearly seen in Figure 4ai,aii, in the case of actuation in 1 M NaOH. Elemental spot analysis for spot 1 indicated a Galinstan-rich phase, whereas spot 3 suggests a platinum gallium alloy with traces of indium and tin and oxygen. Spot 2 was bare platinum as seen in the marked spot and the shiny area in Figure 4ai. Similar alloying effects are observed for Galinstan actuated in 1 M HCl as seen in Figure 4bi,bii. The resulting elemental composition for three analyzed spots are similar to those seen for NaOH, except for spot 3 where the oxygen composition for HCl-actuated droplet was significantly higher than NaOH. Overall, the XRD plots showed no difference in peak intensity or shifts and as such are inconclusive even though the alloying is clearly present. In principle, GLMAs in ambient conditions will wet the metal substrate and adhere due to the inherently forming oxide layer. However, in the presence of strong reducing agents like NaOH and HCl, the oxide skin is continuously reduced, and the post-actuation alloying strongly relies on the intermetallic bonding between GLMAs and MEs. Two of the most commonly used conductive metals (Au: 4.52 × 10^7^ S m^–1^, Pt: 0.95 × 10^7^ S m^–1^ at 293 K)^47^ have a strong tendency to react with Galinstan and cannot be used for applications involving direct contact with GLMs. Interestingly, 100 nm Ti electrodes show excellent alloying resistance to an actuated Galinstan droplet and have no visual alloying effect (Figure 5). However, actuating a droplet of Galinstan on Ti electrodes requires voltages in excess of −15 V irrespective of 1 M NaOH or 1 M HCl due to the poor electrical conductivity of Ti (0.05 × 10^7^ S m^–1^ for 100 nm Ti; Table 1). Passing voltages greater than −15 V also result in anodizing effects, such as the formation of an oxide layer on the bare titanium surface. The anodization effect can be seen for a Galinstan droplet actuated in 1 M NaOH (Figure 5ai) represented by brown and violet, and brown and blue for 1 M HCl (Figure 5bi). For both the cases (NaOH and HCl), gallium, indium, and tin were not detected in EDX, confirming the alloying-barrier effect of titanium. Moreover, the elemental spot analysis showed about 7 wt % of titanium, with the rest denoting soda lime glass and ∼30 wt % or higher amounts of oxygen from the anodization effect. The XRD plot showed a sharp peak for bare Ti; however, on actuated samples, that peak was lost and the plots were rather scattered, indicating a change in the orientation of Ti. We also investigated the effect of a 2.5-μm-thick Ti film with measured conductivity of 0.19 × 10^7^ S m^–1^; however, actuating Galinstan in 1 M NaOH or 1 M HCl needed voltages greater than −6 V, which also resulted in anodization of the actuated area. We also studied the effect of sputtering a thin layer of Ti as an alloying-barrier layer on top of Au electrodes, where the underlying Au would provide the electrical conductivity and Ti would be the alloying-barrier layer. However, the main challenge was the adhesion of Ti on Au, which easily detaches upon actuation of liquid metals (see Figure S2). To gain more insight into more MEs in direct contact with Galinstan, we further studied the effect on Ni and WTi electrodes. In the case of 100 nm Ni on 20 nm Ti electrodes, the alloying of Galinstan is dominant in both 1 M NaOH and 1 M HCl (Figure 6a,b). The elemental analysis for Galinstan actuated in NaOH (Figure 6ai,aii) shows the presence of a Galinstan-rich phase (spot 1), bare nickel thin film (spot 3) with oxygen, and spot 2 indicating soda lime glass residue, which was due to a glass slide used to calibrate the probe height being accidentally crushed on the alloyed sample before measurements. Similar elemental composition is observed for Figure 6ai,aii for spots 1 and 2, except for spot 3 that was analyzed at the interface and confirmed the presence of nickel and Galinstan. XRD peaks for the alloyed and reference Ni electrode are in the same 2-θ range with no significant difference in their intensities. While the XRD plots are inconclusive as also seen for platinum, the post-actuation electrode degradation is on par with the metals discussed earlier and confirms that Ni electrodes cannot be used for direct-contact applications with GLMAs. WTi electrodes (100 nm) showed resistance to alloying and electrode surface degradation in 1 M HCl. However, major degradation was observed for a Galinstan droplet actuated in 1 M NaOH (Figure 7a,b). Optical and electron microscopy images for the WTi electrode in 1 M NaOH (Figure 7ai,aii) post Galinstan actuation shows similar effects to those observed for Ti (Figure 5a) with no alloying. However, it is worth noting that the elemental amount of W is significantly less in spot 1 (5.5 wt %), which indicates dissolution of W in NaOH medium. The elemental analysis for both NaOH (except spot 1) and HCl confirmed the presence of the WTi alloy with oxygen possibly from anodization. XRD plot shows slightly left shifted peaks for the alloyed electrodes in comparison to the reference. Although tungsten (W) is electrically conductive (1.89 × 10^7^ S m^–1^ at 293 K),^47^ its conductivity drops to 0.15 × 10^7^ S m^–1^ at 293 K^49^ when doped with Ti. Lower conductivity necessitates applying higher potentials (−10 to −25 V) to actuate Galinstan.

**Table 1:** Comparison of the Effect of Actuating a Droplet of Galinstan on Various Metal Electrodes and Alloying-Barrier Coating in the Presence of 1 M NaOH and 1 M HCl along with the Measured Electrical Conductivity in Relation to the Reported Work and Electrode Preparation Cost^a^

metal type	1 M NaOH	1 M HCl	conductivity (S·m^–1^) at RT measured	conductivity (S·m^–1^) at 293 K in the literature	cost (€)	comment
gold	––	––	2.3 × 10^7^	4.52 × 10^7 57^	4.8	not suitable for actuating Galinstan, instantaneous alloying
platinum	––	––	0.51 × 10^7^	0.95 × 10^7 ^^57^	6.9	not suitable for actuating Galinstan, similar alloying as seen for the gold electrode
titanium	+	–	0.05 × 10^7^	0.17 × 10^7^^58^	16 (100 nm)	no alloying, anodization effect observed since >20 V needed to actuate Galinstan droplet; thicker Ti deposition is expensive, time consuming (4 h) and needs >−6 V for actuation
			*0.19 × 10^7^ (*2.5 μm layer)		74 (2.5 μm)	
nickel	––	––	0.16 × 10^7^	0.15 × 10^7 ^^57^	9.8	not suitable for actuating Galinstan, instant alloying, severe electrode degradation
tungsten–titanium	+	+	0.84 × 10^7^	1.4 × 10^7 ^^59^	22.2	tungsten dissolves in 1 M NaOH on passing voltage, no severe alloying in 1 M HCl; >20 V needed to actuate Galinstan droplet due to poor electrical conductivity
polypyrrole on gold	++	++	0.64 × 10^7^	NA		excellent coating against alloying, stable in basic conditions; polypyrrole can be selectively redeposited; Galinstan actuation at –5 V

a –−) Severe alloying and surface degradation, (−) alloying, (+) resistant to alloying, (++) excellent resistance to alloying and surface degradation.

The above-presented results conclude that many of the most commonly sputtered metals ranging from Au, Pt, and Ni, to Ti and WTi cannot be used in direct contact with GLMs due to their tendency to alloy (Au, Pt, Ni) and undergo degradation on LM actuation (specifically Ti, WTi), irrespective of their electrical conductivities (Table 1). The challenge of alloying and degradation of such electrodes limits their application in direct contact with Galinstan. We overcome this challenge by electrodepositing the conductive polymer polypyrrole on etched Au electrodes and achieved nonalloying behavior without compromising on the actuation performance.

### *In Situ* Deposition of Polypyrrole on Au Electrodes

**Figure 8:**
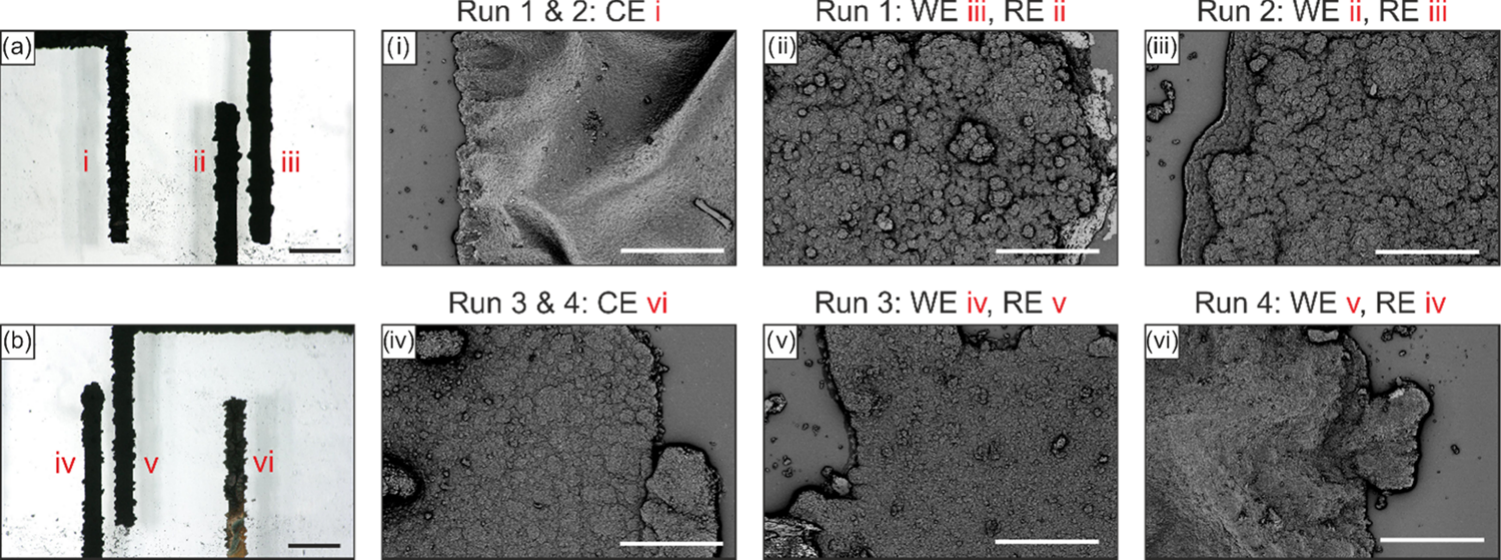
Electrodeposition of PPy on etched Au electrodes with a three-electrode cell via cyclic voltammetry. (a, b) Optical microscopy image of electrodeposited PPy on etched Au electrodes i–vi (captured at 10° tilt for better visualization; black scale bar: 500 μm) along with the corresponding morphology captured via electron microscopy (white scale bar: 50 μm). Labels at the top indicate the respective electrodes used as the working electrode (WE), reference electrode (RE), and counter electrode (CE) for each electrodeposition run.

PPy was electrodeposited on six etched Au electrodes (*E*_i_–*E*_vi_) by casting a drop of pyrrole solution (Figure 8a,b) in four runs by applying voltage from 0 to 1000 mV at 20 mV·s^–1^ scan rate via half-cycle CV while recording the resulting current. In the first run, where all electrodes are bare-etched Au without any alloying barrier, *E*_iii_ was set to the working electrode (WE), *E*_ii_ was the reference electrode (RE), and *E*_i_ was the counter electrode (CE). With this setup, PPy was electrodeposited on *E*_iii_ as presented in Figure 8aiii. In the second run, we switched between WE and RE and the previously electrodeposited PPy electrode *E*_iii_ was set as the RE, whereas bare-etched Au electrode E_ii_ was the WE, while keeping the *E*_i_ as the CE. By switching the electrodes and using a PPy-deposited electrode as the RE, we avoid the need to have an additional Au electrode that would only function as the RE and alloying of this Au electrode that would negatively affect the *in situ* actuation. The cyclic voltammetry plot suggests that using an electrodeposited PPy electrode as a RE in run 2 results in a negative shift in the recorded oxidation peak, from 800 to 900 mV for run 1 to 700 to 850 mV for run 2 (Figure S1a). The thickness of the electrodeposited PPy in the first run (11.8 ± 2.9 μm) was slightly lower than that deposited in the second run (13.5 ± 2.2 μm). Such slight difference confirms that setting the bare-etched Au electrode or the electrodeposited PPy electrode to the RE makes no significant change in the final thickness of the deposited PPy. The recorded current for runs 1 and 2 exceeds the set limit of 900 μA on our three-electrode cell and hence shows a constant line instead of an oxidation peak. PPy electrodeposited on etched Au electrodes in another reported work showed oxidation peaks in the range of 1500 μA with Ag/AgCl as the RE.^48^ This suggests that the oxidation could also peak above 900 μA in our case, if not limited by our system. The resulting morphology of PPy on *E*_ii_ and *E*_iii_ has cauliflower-like morphology and is similar to that reported in the literature (Figure 8aii,aiii).^50^ Remarkably, owing to some side-reactions, some electrodeposition of PPy occurs on *E*_i_, i.e., the CE, in runs 1 and 2. In a three-electrode cell, the CE is primarily responsible to pass current between itself and the WE to complete the circuit, which could be a major reason for the stray electrodeposition of PPy.^51^ This stray deposition of PPy on the CE provides an additional alloying-barrier electrode for *in situ* actuation of LMs. However, the deposited layer is inhomogeneous and is much thicker (25.1 ± 6.0 μm) than the intentionally deposited PPy on *E*_ii_ and *E*_ii_. We further observed that this stray deposition of PPy on the CE is unpredictable as in some cases, no PPy is deposited on the CE in spite of using the same parameters. Similarly, run 3 included *E*_iv_ as the WE, *E*_v_ as the RE and *E*_vi_ as the CE, where all bare-etched Au electrodes were subjected to a potential scan, resulting in electrodeposition of PPy on *E*_iv_ (Figure 8biv). Consequently for run 4, the PPy-deposited *E*_iv_ was the RE, *E*_v_ was the WE, and *E*_vi_ was the CE, resulting in deposition of PPy on *E*_v_ as seen in Figure 8bv. The recorded CV plot shows an oxidation peak for run 3 between 900 and 950 mV at 600 μA, with a much broader peak compared to run 4, from 800 to 1000 mV at 500 μA (Figure S1b). The higher oxidation peak at 600 μA for run 3 was in accordance with a thicker electrodeposited PPy layer (7.6 ± 2.5 μm) compared to run 4 at 500 μA (5.4 ± 0.8 μm). This is in line with the underlying fact that the corresponding current for a given oxidation peak affects deposition of PPy. It is further attested by the much thicker deposited PPy layer for runs 1 and 2, where the current exceeded the set limit, and 5 μm increase in thickness was observed comparing runs 3 and 4. Since runs 1 and 2 are conducted with the fresh PPy solution, the resulting imbalance in ion concentration and side-reaction residues could affect the deposition process in runs 3 and 4, where the current and the thickness of PPy reduced. The electrodeposited PPy for runs 3 and 4 shows a similar cauliflower-like morphology (Figure 8biv,bv) and *E*_vi_ is similarly deposited with stray PPy as *E*_i_ (14.5 ± 3.7 μm), confirming the observations as noted earlier. While techniques such as chemical oxidative polymerization, ultrasonic assisted polymerization, and electrospinning allow the electrodeposition of pyrrole to PPy, electrochemical polymerization (electrodepositon) offers the unique advantage of in situ polymerization, which allows for better control over the coating thickness and the freedom to deposit PPy coatings on electrodes by simply drop-casting or injecting pyrrole solution inside microfluidic channels with electrodes.^47,52−54^ Unlike standard cyclic voltammetry cells with a continuously stirred solution and clean reference and counter electrodes, our in situ deposition approach is more robust for the scaled-up applications with an array of electrodes. In that sense, PPy is deposited on an array of electrodes of the same metal, only by switching among electrodes and without designated reference and counter electrodes.

**Figure 9:**
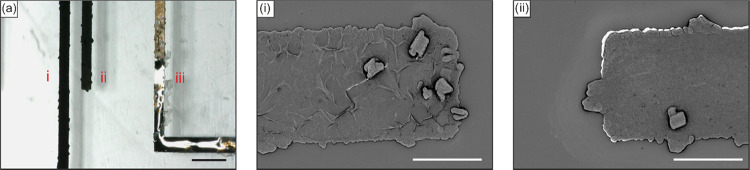
(a) Alloying-barrier properties of electrodeposited PPy on etched Au (electrodes i and ii) post-actuation of a droplet of Galinstan in 1 M NaOH at −3 V with bare-etched Au electrode iii in comparison exhibiting alloying (captured at 10° tilt for better visualization; black scale bar: 500 μm). (i, ii) Morphology of the PPy electrode surface post-actuation, confirming the absence of any alloying residues (white scale bar: 100 μm).

With the etched Au electrodes successfully electrodeposited with PPy, we further tested alloying-barrier property by actuating a droplet of Galinstan *in situ* in 1 M NaOH at −3 V (Figure 9). We observed no alloying of Galinstan on the PPy electrodes (*E*_i_, *E*_ii_) post-actuation for 10 cycles, and *E*_iii_ (CE) showed significant alloying on the etched Au electrode that had no stray deposition of PPy. Electron microscopy images of the post-actuation PPy electrodes *E*_i_ and *E*_ii_ confirm the absence of any Galinstan alloying on the surface and the adhesion of PPy to the etched Au electrode (Figure 9ai,aii). The optimal voltage range (−3 to −5 V) for actuating Galinstan was low since higher voltages result in excessive hydrolysis and further generation of bubbles from the underlying Au electrode. These bubbles tend to delaminate the PPy layer even though the adhesion is enhanced owing to the additional roughness due to electroplating and etching. This suggests that the electrodeposited PPy on etched Au electrodes shows excellent alloying-barrier properties toward LMs and is stable against delamination due to hydrolysis in the optimal voltage range. Polypyrrole is a conjugated polymer with alternating single and double bonds, resulting in sp^2^ hybridization. It has been shown that sp^2^ bonded materials (graphene, carbon ink, conducting polymers―PEDOT/PSS, polyaniline, etc.) are inert and resistant to alloy formation with gallium liquid metals.^16,30,55^ While the exact mechanism is unclear, it is believed that liquid metals are unable to form an interactive bond with the carbon chains in PPy, in comparison to the strong metal bonds between liquid metals and conventional metals. Moreover, among conductive polymers with high electrical conductivity and long-term stability, PPy shows excellent potential stability (very low potential drift) over 30 days in comparison to PEDOT, polyaniline, and poly(3-octylthiophene).^56^ This makes PPy an optimal candidate to conduct in situ deposition. We also compared the performance of PPy-coated electrodes against alloying-barrier coatings reported in the literature in Table S2.

**Figure 10:**
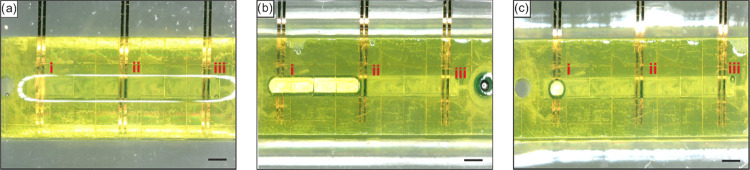
*In situ* actuation of a Galinstan plug in 1 M NaOH at −5 V inside a 500 μm microchannel (scale bar: 500 μm). (a) Electrodeposited PPy on etched Au electrodes (ii and iii) in comparison with bare-etched Au electrodes (i). (b) 3 mm Galinstan plug movement from right to left over the PPy-coated and bare Au electrodes. (c) Post Galinstan plug removal indicating significant alloying on bare-etched Au electrodes (i) and no alloying on PPy-electrodeposited electrodes (ii and iii).

To test the extent of applicability of the electrodeposited PPy electrodes, we 3D printed a straight-channel chip with 500 μm width microchannel with inlet and outlet reservoirs on bare-etched Au electrodes patterned on a glass slide. Electrodes *E*_ii_ and *E*_iii_ were electrodeposited with PPy *in situ* as seen in Figure 10a. *E*_i_ was not coated with PPy to directly test the alloying-barrier property in comparison with bare-etched Au electrodes. A 3 mm long Galinstan plug was injected and then actuated *in situ* via CEW at −5 V from *E*_iii_ to *E*_i_ in 1 M NaOH, and no alloying was observed for *E*_ii_ and *E*_iii_ where PPy was electrodeposited (Figure 10b). However, the bare-etched Au electrodes (*E*_i_) were instantly alloyed with Galinstan and the alloyed LM could not be removed (Figure 10c). We used −5 V instead of −3 V due to the larger plug size, which furthermore induced hydrolysis and detachment of the PPy layer for longer actuation time. We additionally tested the actuation of a Galinstan plug in a 3D printed chip with all etched Au electrodes coated with PPy. The plug was actuated over all of the PPy-coated electrodes by applying 5 V square wave (Video S1). The PPy electrodes are stable for 150 continuous actuations, until the PPy coating over one electrode detached due to hydrolysis (Video S2). We believe that the PPy coating will be stable for a longer duration than 150 actuations if smaller LM plugs are used, which in turn allows the use of lower voltages (−1 to −2 V) that will significantly reduce hydrolysis. In general, for electrodepositing PPy, in addition to pyrrole, we need both an oxidant and a solvent, in our case KCl and distilled water, respectively. Changing the concentration of either of these influences the deposited PPy morphology, thickness, and stability.^52^ Literature reports the oxidation peaks of PPy are in the range of 600–900 mV, and hence we run the scan till 1000 mV to encounter an unintended shift in the oxidation peak owing to an imbalance in the pyrrole solution. We opted 20 mv/s as our scan rate since lower scan rates result in the oxidation of pyrrole to PPy over a long period of time, thus depositing a thicker PPy coating, whereas faster scan rates result in thin PPy coatings in the nanometer range.^47^ Based on these parameters, PPy-coated electrodes of 5–10 μm at 20 mV/s show alloying resistance for over 150 actuations. In an attempt to provide a durable alloying-barrier layer and a reproducible deposition of PPy, the detached PPy layer can be rinsed out from the microchannel and fresh pyrrole solution be injected and electrodeposited to obtain PPy-coated electrodes. This *in situ* deposition of PPy is localized and allows unrestricted freedom in obtaining alloying-barrier electrodes for LM–ME contact applications.

**Figure 11:**
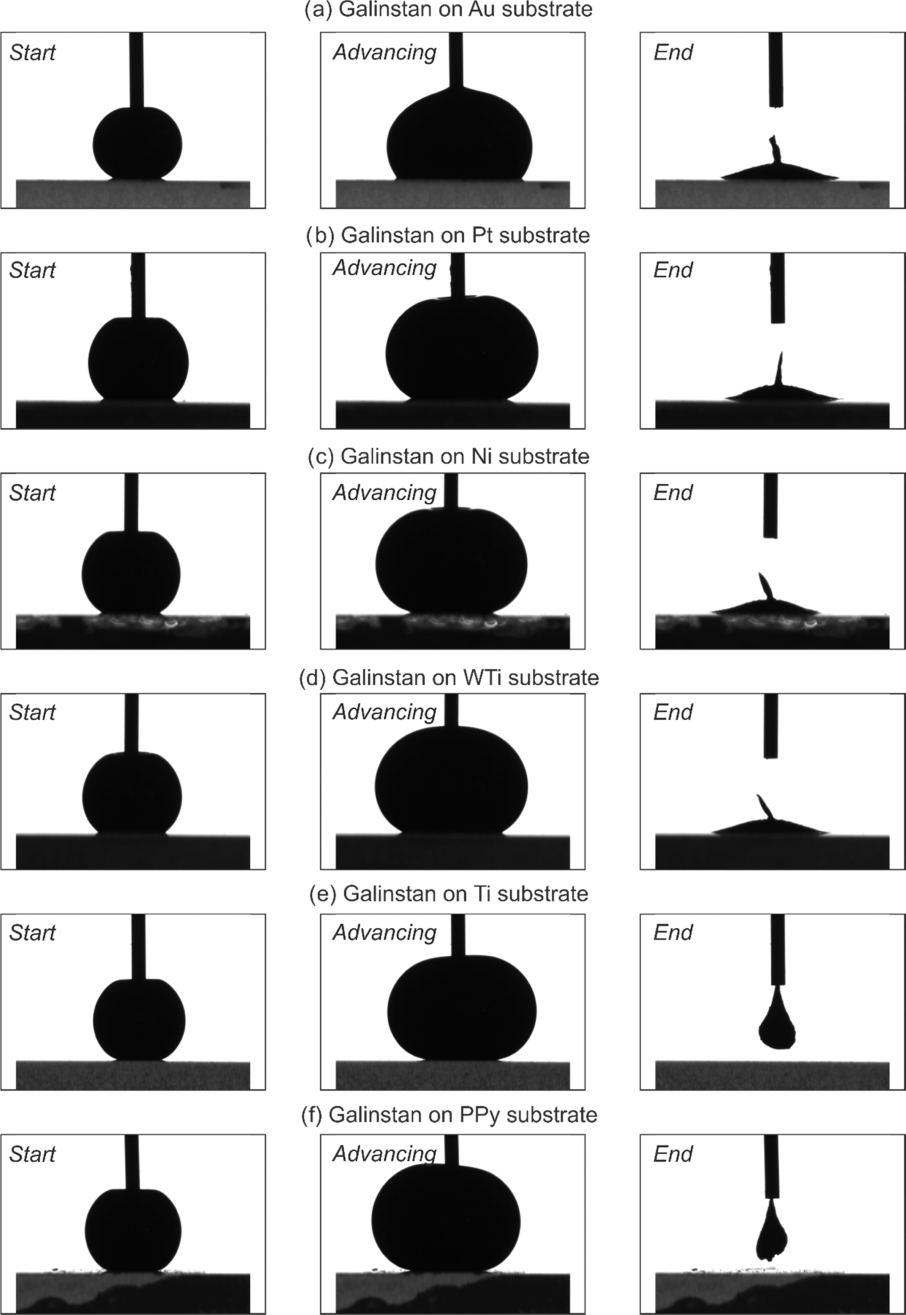
Still shots of a Galinstan droplet on different substrates: (a) Au, (b) Pt, (c) Ni, (d) WTi, (e) Ti, and (f) PPy at the start (left column), during advancing (center column), and at the end (right column) of recorded ARCA measurements. The substrates from panels (a–d) show residual Galinstan at the end indicating alloying, whereas panels (e, f) show no Galinstan residue indicating alloying-barrier properties. The needle tip with an OD of 0.3 mm assists as a scale bar.

Wetting characterization of Galinstan on different substrates studied in this work confirmed the alloying behavior of Au, Pt, Ni, and WTi indicated by strong adhesion due to metal–metal bonding resulting in pinning of Galinstan on the substrates denoted by the left over Galinstan residue (Figure 11a–d). Interestingly, Galinstan droplet on Au, Pt, Ni, and WTi showed extremely low receding contact angles (Table S1) in the range of 5–11°, indicating strong affinity of Galinstan to wet these substrates (Video S5). Both Ti and PPy showed excellent alloying-barrier properties, indicated by the completely receded Galinstan droplet leaving behind no residue (Figure 11e,f and Videos S3 and S4) and supported by the receding contact angles of 74.82 and 76.42°, respectively (Table S1). Moreover, these measurements were conducted in atmospheric conditions without the presence of 1 M NaOH or 1 M HCl, indicating the significance of the Galinstan droplet with the oxide skin, contributing to pinning and adhesion to Au, Ni, Pt, and WTi and not to Ti and PPy. For actuation purposes where an electrolyte is present, the oxide skin does not exist; however, the interactions between the liquid metal and the electrode metal substrate show different results as seen earlier.

## Conclusions

In this work, we investigated the effect of an actuated Galinstan droplet on various electrodes ranging from Au, Pt, and Ti to Ni and WTi in aqueous mediums of 1 M NaOH and 1 M HCl. Au, Pt, and Ni electrodes undergo instantaneous alloying, while Ti and WTi show good alloying resistance, although they are susceptible to electrode surface degradation and require high voltages exceeding −15 V to actuate a millimeter-long Galinstan plug. For low-voltage applications, we presented a convenient approach to obtaining alloying-barrier electrodes by electrodepositing PPy via cyclic voltammetry on electroplated and etched Au electrodes. We show that the electrodeposited PPy has excellent alloying resistance to Galinstan and does not detach from the underlying Au electrode surface. We demonstrated this approach by 3D printing of a microfluidic chip directly on top of the Au-patterned glass substrate and performing the PPy deposition step *in situ*. We showed the alloying-barrier property and adhesion stability of PPy by actuating a Galinstan plug in 1 M NaOH via CEW at −5 V for over 150 actuations. We also tested the reproducibility and durability of this process. We believe that this work will be instrumental to further development of applications using liquid metals in direct contact with metal electrodes as commonly found in, e.g., MEMS, microfluidics, or microactuators, where alloying-free movement of liquid metals is essential.

## Supporting Information Available

Cyclic voltammetry plots for PPy deposition on etched Au electrodes (Figure S1); effect of a Galinstan droplet actuated on Ti–Au–Ti electrodes in 1 M NaOH and 1 M HCl (Figure S2); advancing and receding contact angles of Galinstan on different substrates studied in this work (Table S1); and comparison of the reported barrier coatings against LMs in the literature vs PPy-coated electrodes shown in this work (Table S2) (PDF)
Actuation of a Galinstan plug in 1 M NaOH via CEW at 5 V by applying a square wave (Video S1) (MP4)
Detachment of PPy coating over one electrode after more than 150 actuations (Video S2) (MP4)
ARCA measurement of Galinstan on the PPy-coated Au electrode (Video S3) (MP4)
ARCA measurement of Galinstan on the Ti electrode (Video S4) (MP4)
ARCA measurement of Galinstan on the Au electrode (Video S5) (MP4)
Arduino Uno code used for programming the square wave for CEW actuation of Galinstan (ZIP)
