# Supplementary material for: In Situ Actuators with Gallium Liquid Metal Alloys and Polypyrrole-Coated Electrodes
Source: ACS Appl Mater Interfaces. 2023 Feb 8;15(7):10109–22. doi: 10.1021/acsami.2c17906 (PMC9952059; doi:10.1021/acsami.2c17906)
Supplement: Supplementary file 1 — am2c17906_si_001.pdf [file am2c17906_si_001.pdf]

Supporting Information for Publication

In situ actuators with gallium liquid metal alloys and polypyrrole-coated electrodes

Sagar Bhagwat[1], Andreas Goralczyk[1], Manuel Luitz[1], Lathif Sharieff [1], Sebastian Kluck[1], Ahmed Hamza[1], Niloofar Nekoonam[1], Frederik Kotz-Helmer[1,2], Pegah Pezeshkpour[1,2] and Bastian E. Rapp[1,2,3]*

[1] Laboratory of Process Technology, NeptunLab, Department of Microsystems Engineering (IMTEK), University of Freiburg, Georges-Köhler-Allee 103, 79110 Freiburg, Germany.

[2] Freiburg Materials Research Center (FMF), University of Freiburg, Stefan-Meier-Straße 21, 79104 Freiburg, Germany.

[3] FIT Freiburg Center of Interactive Materials and Bioinspired Technologies, University of Freiburg, Georges-Köhler-Allee 105, 79110 Freiburg, Germany.

*pegah.pezeshkpour@neptunlab.org

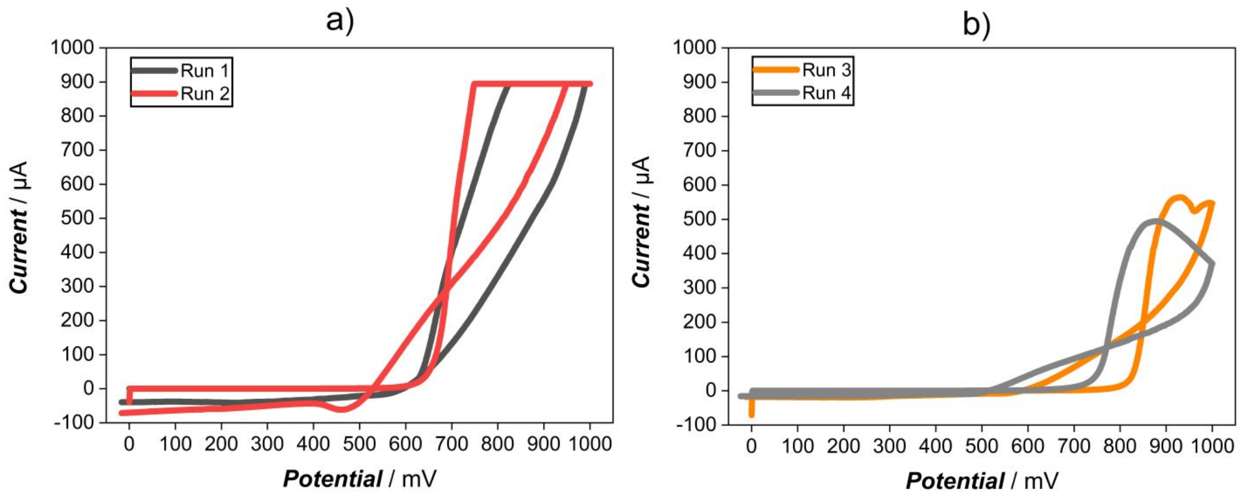

Figure S1: Cyclic Voltammetry plots for electrodeposition of PPy on etched Au electrodes for a potential scan from 0 – 1000 mV at 20 mV·s^{-1}. a) Cyclic Voltammetry plot for Run 1 (WE iii, RE ii, CE i) and Run 2 (WE ii, RE iii, CE i) with the corresponding oxidation peaks between 650 - 900 mV. b) Potential vs. Current plot for Run 3 (WE iv, RE v, CE vi) and Run 4 (WE v, RE iv, CE vi) with the corresponding oxidation peaks between 750 - 950 mV.

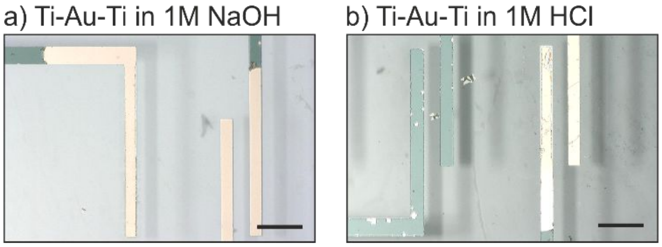

Figure S 2: Effect of a Galinstan droplet actuated at -1 V on 100 nm Gold electrodes with 20 nm Titanium adhesion layer sputtered with 20 nm Titanium layer as an alloying barrier coating in presence of 1 M NaOH (a) and 1 M HCl (b) resulting in detachment of the titanium layer via optical microscopy images (captured at 10˚ tilt for better visualization; black scale

Table S 1: Overview of the advancing contact angles (average and standard deviation of 3 measurements) and the corresponding receding angles of a Galinstan droplet on different substrates characterized via ARCA measurements.

Substrate	Advancing Angle (˚)		Receding Angle (˚)
	Average	Std. Dev.	
Au	151.09	1.04	5.49
Pt	151.39	2.24	7.84
Ni	158.1	1.93	7.94
WTi	150.17	1.61	11.82
Ti	162.52	1.54	74.82
PPy	156.32	2.95	76.42

Table S 2: Comparison of different alloying barrier coatings to the PPy coating shown in this work in terms of actuation and performance

Substrate	Alloying barrier type	Actuation of liquid metal	Performance	References
Ag NPs	SWCNT	No	Resistance stable over 300 h for EGaIn in direct contact with SWCNT	Oh *et al.*[1]
Ti	Diamond coating	No	Diamond coated Ti showed non-stick behavior and excellent resistance to Galinstan when used as an electrode or switch for 3000 h	Handschuh-Wang *et al.*[2]
Al	PEDOT:PSS/GO composite	No	Change in electrical resistance of barrier coating was below 0.4 % on exposure to Galinstan for 10 days	Shin *et al.*[3]

Soft elastomer	NeverWet®	No	Spray coating of NeverWet to obtain non-stick (oxide-phobic) surfaces to avoid LM adhesion; coating stable for over 30 months	Joshipura *et al.*[4]
Au	PPy	Yes	PPy alloying barrier coating stable for over 150 continuous actuations of a Galinstan plug at -5 V in 1M NaOH; PPy coating shows repellence towards bare Galinstan droplet (with oxide skin)	This work

Supplementary Videos

We have included the videos in .MP4 format

Video S 1: Actuation of a Galinstan plug in 1 M NaOH via CEW at 5 V by applying a square wave

Video S 2: Detached PPy coating over one electrode after more than 150 actuations

Video S 3: ARCA measurement of Galinstan on PPy coated Au electrode (5x sped up)

Video S 4: ARCA measurement of Galinstan on Ti electrode (5x sped up)

Video S 5: ARCA measurement of Galinstan on Au electrode (5x sped up)

Arduino Uno code used for programming the square wave for CEW actuation of Galinstan (.ino)

References

(1) Oh, E.; Kim, T.; Yoon, J.; Lee, S.; Kim, D.; Lee, B.; Byun, J.; Cho, H.; Ha, J.; Hong, Y. Highly Reliable Liquid Metal-Solid Metal Contacts with a Corrugated Single-Walled Carbon Nanotube Diffusion Barrier for Stretchable Electronics. *Adv. Funct. Mater.* **2018**, *28* (51), 1806014. https://doi.org/10.1002/adfm.201806014.

(2) Handschuh-Wang, S.; Wang, T.; Zhu, L.; Xu, Y.; Huang, L.; Gan, T.; Tang, Y.; Zhou, X. Corrosion-Resistant Functional Diamond Coatings for Reliable Interfacing of Liquid Metals with Solid Metals. *ACS Appl. Mater. Interfaces* **2020**, *12* (36), 40891–40900. https://doi.org/10.1021/acsami.0c09428.

(3) Shin, D.; Baek, S.; Song, H.; Lee, J. I.; Kang, G. Sliding Interconnection for Flexible Electronics with a Solution-Processed Diffusion Barrier against a Corrosive Liquid Metal. *Adv. Electron. Mater.* **2019**, *5* (10), 1900314. https://doi.org/10.1002/aelm.201900314.

(4) Joshipura, I. D.; Ayers, H. R.; Castillo, G. A.; Ladd, C.; Tabor, C. E.; Adams, J. J.; Dickey, M. D. Patterning and Reversible Actuation of Liquid Gallium Alloys by Preventing Adhesion on Rough Surfaces. *ACS Appl. Mater. Interfaces* **2018**, *10* (51), 44686–44695. https://doi.org/10.1021/acsami.8b13099.
